# AceTree: a tool for visual analysis of *Caenorhabditis elegans *embryogenesis

**DOI:** 10.1186/1471-2105-7-275

**Published:** 2006-06-01

**Authors:** Thomas J Boyle, Zhirong Bao, John I Murray, Carlos L Araya, Robert H Waterston

**Affiliations:** 1 Department of Genome Sciences, University of Washington, Seattle, WA 98195, USA

## Abstract

**Background:**

The invariant lineage of the nematode *Caenorhabditis elegans *has potential as a powerful tool for the description of mutant phenotypes and gene expression patterns. We previously described procedures for the imaging and automatic extraction of the cell lineage from *C. elegans *embryos. That method uses time-lapse confocal imaging of a strain expressing histone-GFP fusions and a software package, StarryNite, processes the thousands of images and produces output files that describe the location and lineage relationship of each nucleus at each time point.

**Results:**

We have developed a companion software package, AceTree, which links the images and the annotations using tree representations of the lineage. This facilitates curation and editing of the lineage. AceTree also contains powerful visualization and interpretive tools, such as space filling models and tree-based expression patterning, that can be used to extract biological significance from the data.

**Conclusion:**

By pairing a fast lineaging program written in C with a user interface program written in Java we have produced a powerful software suite for exploring embryonic development.

## Background

The invariant lineage of the nematode *C. elegans *[1] can potentially be exploited to capture detailed information on the location and timing of expression for the genes expressed in the early embryo. In addition, changes in the lineage resulting from mutations or RNAi knockdowns of gene function can provide functional information about genes. To use the lineage with high throughput, one must capture images in sufficient detail and subject those images to automated lineaging.

Bao et al[2] described a procedure for generating lineages automatically. A ubiquitously expressed histone-GFP fusion protein is used to label nuclei in the developing embryo. Images sets are captured once a minute by a confocal microscope, with each set containing up to 35 focal planes through the full depth of the embryo. The program StarryNite analyzes the images to locate all the nuclei at each time point and to establish the linkage of nuclei from time point to time point. The resulting annotation, which implicitly establishes the lineage of the embryo, is written to a series of files, called the nuclei files, one for each time point.

A separate program, AceTree, was written to facilitate viewing, editing and interpretation of the StarryNite output and is described here. This program is separate from StarryNite, with distinct requirements. StarryNite was written in C with a minimal user interface because of the computationally intensive character of automated lineage extraction. The editing and interpretation tools required for AceTree require robust and portable user interfaces and the ability to develop and test new tools rapidly, making Java a logical choice. In addition, AceTree can be used without StarryNite output as an image viewer and manual lineaging tool, although it is not currently optimized for the latter task.

Three other software packages exist that link image series and lineage trees. The most complete of these is SIMI BioCell, which is optimized for manually lineaging of 4D differential-interference-contrast (DIC) image series and has been used effectively to demonstrate the striking insights that can be obtained by lineage analysis[3]. Angler was developed by the developers of AceDB and is directed mainly at viewing and interpreting lineaged series with more reference to information stored in WormBase[4]. Virtual Wormbase has both an educational goal and a research goal and embeds the idea of simulating the development process[5]. These were all designed to deal with 4D DIC image series and thus are not optimized for the specifics of GFP-histone image series.

We chose to develop a new program, AceTree, rather than attempt to adapt an existing program for several reasons. The new program could be optimized for viewing fluorescence images, including "second color" images used to track gene expression throughout embryogenesis. The key data structure of AceTree is identical with the nuclei files produced by StarryNite, which facilitates the expected co-evolution of the programs as the project matures. In addition, AceTree is being made available as an open source package supporting all major PC operating systems.

## Implementation

Since computation speed is unlikely to be an issue in the user interface oriented AceTree, a high-level language solution was sought. Many biological analysis programs with user interface aspects are written in Java and that language received the top consideration. Two components were needed: a package for handling tiff images and a way to produce interactive trees. ImageJ[6] met the first requirement and provided extra features some of which have since been used to assist in handling the image series as they come from the microscope and to produce and view movies developed from images arising in AceTree. The Java class known as DefaultMutableTreeNode contains all the normal features of a tree data structure and the JTree class provides a convenient graphical user interface for it.

AceTree is written in Java version 1.4.2. For 3D representations the Java3D module is required. Image operations of AceTree are derived from ImageJ and the ij.jar file is required. Development is carried out in the open source Eclipse Platform [7,8]. For source distribution an Ant [9] build.xml file is provided. The program is packaged as a jar file and has been tested on conventional workstations using three different operating systems: linux; Mac OS X; windows.

## Results and discussion

### Program components

**Figure 1:**
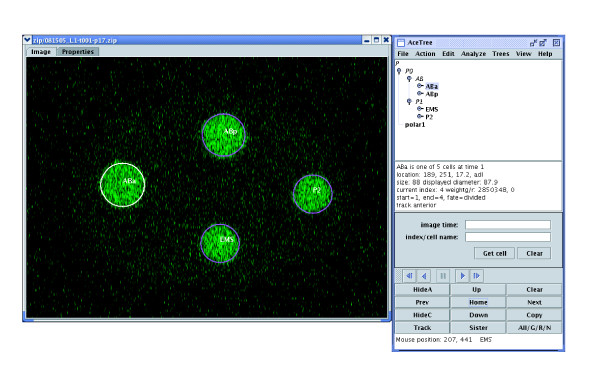
**AceTree main control and ImageWindow**. The image window shows the annotated data for the four-cell stage embryo at the first time point of the series. The main control shows the JTree representation with cell ABa highlighted. The four founder cells, ABa, ABp, EMS, P2 can be seen in both the image window and the tree view. The tree also shows that a polar body has been found at this time point, called polar1. However it is not visible in plane 17, the plane shown in the image window.

The normal operating mode of AceTree is shown in Figure 1 where the process of opening a set of data is complete and the user is viewing annotated images in the ImageWindow and navigating among them using the main control. The title bar of the ImageWindow contains a coded part, "t001-p17" which tells the user that this image is from time point 1 plane 17 of the image series under study, "081505". The underlying tiff image contains only a single gray scale "haze", with strong (whiter) clusters corresponding to the green fluorescent protein histone fusions. AceTree shows this in the green plane of an RGB image and has added annotations.

The imaging protocol has in this case captured the four-cell stage of the embryo in the first time point. The basic image annotation consists of blue circles around the nuclei, which have been modelled as spheres. At any time, one of the cells will have the special designation of "current cell" and will have a white circle, in this case the ABa cell. Cell names are a basic element of the annotation scheme and AceTree offers the user several ways to control which cells carry their names on the displayed images.

The main control, shown on the right in Figure 1, contains the following seven parts:

1. A menu bar some of whose application specific functions will be addressed herein.

2. A "JTree" representation of the lineage where ABa is highlighted.

3. A text display window with information about the current cell being displayed.

4. A control that enables the user to bring up a particular cell at a particular time point.

5. A "movie" control that can be used to automatically sequence through the images tracking the current cell and its descendents.

6. A pad with 12 buttons. These buttons provide considerable control over navigation and the kinds of information shown with the image. The main navigation keys – next, prev, up, down – are mapped to the arrow keys on the keyboard.

7. A text window displaying the position of the mouse when it is within the image.

**Figure 2:**
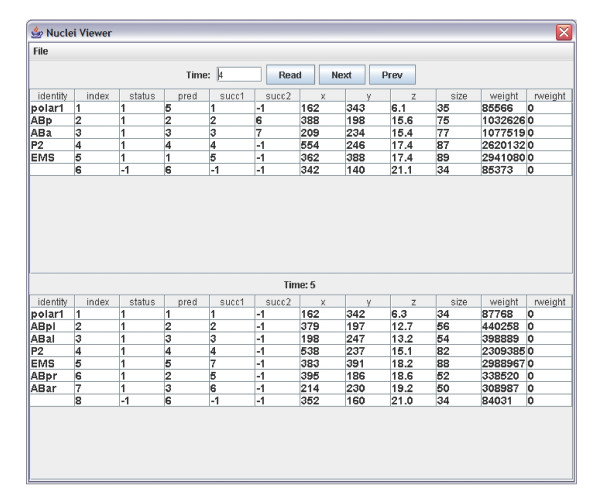
**View of nuclei data**. Nuclei data from time points 4 and 5 are shown. The two divisions that occurred between times 4 and 5 can be traced from the successor flags. Nuclei location, size, and canonical names are also shown. These are the annotations produced by the automated lineaging software.

Although it is not routinely needed during the study of an image series, AceTree via its Analyze..NucleiView menu presents the automated lineaging data structure on which all of the interaction and annotation is based. A sample of this display is shown in Figure 2.

**Figure 3:**
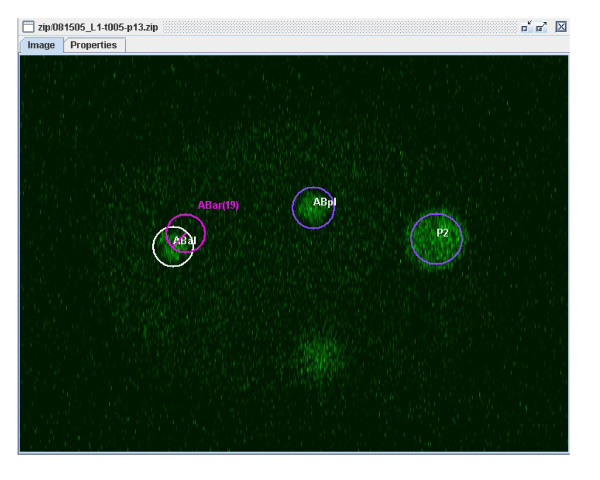
**Tracking to the division of ABa**. The image from plane 13 is annotated to show ABal as the current cell and a ghost representation of cell ABar which is centered at plane 19 and has no natural intersection in this plane. Correct tracking of divisions is a key challenge in automated lineaging.

The figure shows the annotations from times 4 and 5 corresponding to the division of ABa and ABp, the results of which are shown in Figure 3. The identity, x, y, z, and size columns are readily understood as the name, position, and diameter in pixels of the identified nucleus. The pred, succ1, and succ2 columns describe how nuclei at one time point relate to those at adjacent time points. In the absence of a division, succ2 is negative and succ1 contains the index of the continuing nucleus in the next time point. Where a division was detected, succ1 and succ2 point to the two daughter cells in the next time point. In this case, the division of ABa can thus be followed: the two daughters are located at indices 3 and 7 in the nuclei structure at time 5. The propagation of names to daughters follows from the linkage using rules based on the relative locations of the daughters. This is the basis on which AceTree has built its tree representation. The weight and rweight columns contain the summed intensity within the spherical representation of the nucleus for the green and red channel images, respectively.

### Cell tracking

Tracking cells from image to image is a common need in analyzing lineaged image data. The "Next" key on the keypad moves to the next image in time while tracking the "current cell". If the nucleus is moving in the z plane (here the left/right embryonic axis), then AceTree adjusts, choosing the z plane image which is closest to the center of the nucleus. Figure 3 shows the image window when tracking has reached time 5. Tracking can be dismissed permitting the user to follow a sequence of images where the plane of the image remains fixed.

In the first time point following a division, the current cell designation will move to one of the daughters of the divided cell. Here, the new "current cell" is ABal: AceTree has tracked the left daughter of the division of the ABa cell. However, it also shows in cyan the sister cell, ABar. The annotation in cyan advises that the nucleus of ABar is centered in plane 19. Since we are looking at the plane 13 image, the cyan annotation is a "ghost nucleus" and in this case reassures us that the embryo has followed the left/right division pattern suggested by its canonical name. Notice also that in the position previously occupied by ABp we have ABpl: both daughters of AB have divided at the same time,

There are multiple ways to select the current cell while studying an image series. The tree representation is "live". One can expand the tree to a cell of interest and left click on it in the tree to bring up the image at its "birth". Alternatively, one can right click on it to bring it up just before its division or death. The cell selection panel can be used to bring up a given time point. Any circle displayed in the image can be right clicked to make that cell the current cell and the tree display is updated correspondingly.

### Curating the automated lineage

AceTree can be used to curate and edit lineaged data series such as those arising from StarryNite. AceTree accepts as its inputs the image series and the annotation files whose content was shown in Figure 2. If the series is edited, AceTree creates a revised set of annotation files which would be used in subsequent studies of the data. The tools of the Edit menu are provided for these purposes.

**Figure 4:**
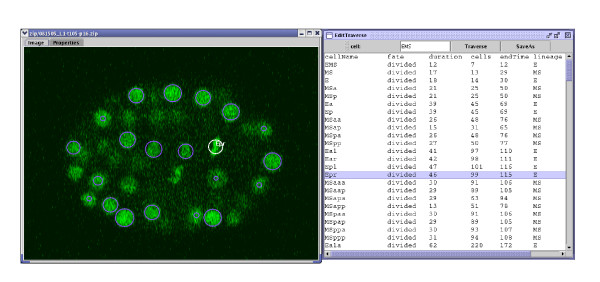
**Lineage curation using EditTraverse**. The EditTraverse list box shows all divisions in the EMS lineage. As the user moves the highlight down the list, the ImageWindow tracks to the corresponding division. Here, cell Epr is about to divide.

Figure 4 shows the EditTraverse tool from that menu along with the ImageWindow when the tool is in use. EditTraverse builds a list of all cell divisions and deaths starting from the cell named in the edit box. As the highlight is moved, the ImageWindow receives the image showing the cell in question just before its division. The user can quickly examine the sequence of events in the time leading up to and following the division to verify that the lineage has correctly tracked this important event. If problems are detected, the EditImage tool can be brought up. That tool makes it possible to add, remove, reposition, resize, and relink cells to correct any errors.

The specific situation shown in Figure 4 shows cell Epr just prior to its division into Epra and Eprp. The image clearly shows the metaphase plate and its orientation for the division along the anterior/posterior axis.

When a lineage is edited in such a way that cell linkage is changed from that in the original nuclei files, the tree representation must be rebuilt and cell naming must be redone. Therefore, AceTree contains the algorithms described by Bao[2] to determine cell names upon division. These include the algorithm for determining the embryonic axes from the four founder cells and the subsequent divisions of ABa and ABp which provide the essential starting point for cell naming. Therefore, it is possible to use AceTree to manually lineage an image series and develop annotation files to be used in subsequent studies of the data although the editing tools are not optimal for such a purpose.

### Viewing embryonic lineage and morphology

**Figure 5:**
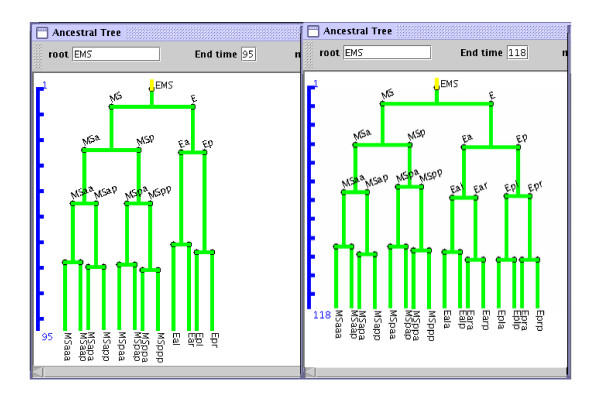
**Comparison of EMS lineages for two developmental series**. The tree on the left is from a wild type embryo; that on the right is from an RNAi treated embryo with the *lit-1 *gene inhibited. This has caused the E lineage to adopt the fate of the MS lineage. The tree view shows this clearly.

The ability to generate automated lineages opens up the possibility of using the lineage as a phenotype in considering developmental changes induced by altered gene function. For this purpose, a tree that tracks the timing of cell divisions is necessary: we call it an ancestral tree. AceTree provides this on its Trees..Ancestral Tree menu. Figure 5 shows two examples of such a tree where the EMS sublineage is shown from a wild type embryo on the left and the corresponding lineage for an embryo where RNAi has been used to inhibit the expression of gene *lit-1 *on the right. The time points from the two different data series were chosen at a point where the total number of cells present is stable for several time points and the AB sublineage consists of 64 cells.

The modified embryo has 4 cells more than the wild type due to an extra division in the E lineage which would normally form the gut. With *lit-1 *function inhibited, the E cells adopt the fate of the MS lineage and the gut fails to form resulting in embryonic lethality[10]. Notice that the E and MS subtrees for the modified embryo are similar to each other and similar to the wild type MS sublineage but not the wild type E sublineage.

**Figure 6:**
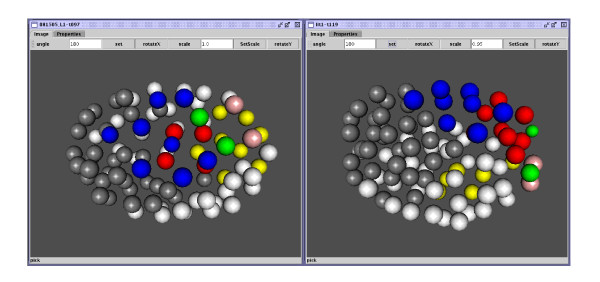
**Developmental comparison using space filling models**. In both images, the time point corresponds to the point where the AB sublineage contains 64 cells. As in Figure 5, the wild type embryo is on the left and the embryo with *lit-1 *inhibited is on the right. The E lineage cells have not migrated to the interior in the RNAi embryo. Cell migration generally has not proceeded in the normal way. These are ventral views with anterior on the left. The nuclei are color-coded so the main lineages can be distinguished: ABa daughters in gray; ABp in white; E in red; MS in blue; C in yellow; D in pink; germ line in green.

Besides tracking the lineage of cells, the annotation scheme used here (see Figure 2) describes the location and size of each nucleus. This raises the possibility of studying the morphology of the embryo in a 3D space filling model representation. AceTree provides this in its View..3D View menu. Figure 6 shows the results of that tool from each of the series discussed above: wild type embryo on the left and *lit-1 *inhibited embryo on the right. Both views have been rotated so one sees the ventral side of the embryo with the anterior on the left. The nuclei are color coded so the main lineages can be distinguished: ABa daughters in gray; ABp in white; E in red; MS in blue; C in yellow; D in pink; germ line in green.

At this stage the four E cells (red) of the wild type embryo have migrated to the interior beginning the process of gut formation. The space filling model of the *lit-1 *inhibited embryo shows the E cells remaining on the outside of the embryo and matching in number the MS cells. Aside from the abnormal gastrulation, the positioning of other sublineages has also been altered. AceTree offers considerable freedom to select the colors of different sublineages and the orientation of the model to explore morphology.

### Tracking gene expression

Tracking the expression of individual genes in time and cellular location is of major interest to developmental biologists. The automated lineaging technique can be used to track expression of genes of interest by introducing a nuclear localized red fluorescent reporter of a gene's promoter activity. The imaging system then collects a parallel set of images in the red channel and the location and size information in the GFP-derived annotations is used to compute the red intensity of each nucleus.

**Figure 7:**
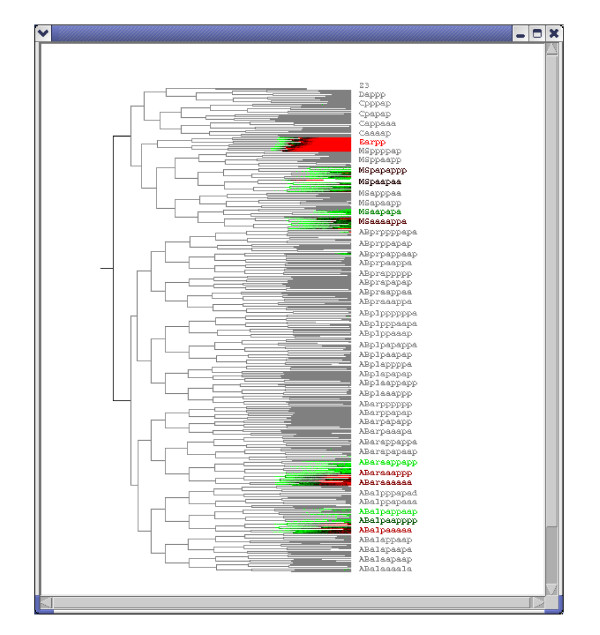
**An overview of the lineage with gene expression shown**. This is a compact representation of the entire lineage of an embryo with the *pha-4 *gene tagged with a red fluorescent protein. The branches are color-coded by the strength of the red expression: gray if there is no expression, generally the case early in the development; green where there is a detectable low level of expression; red where expression is strong. Branch labels are offered for every tenth nucleus which is sufficient to identify the cell groupings where the gene is expressed: strongly in the E lineage, less so in some MS lineages and in some daughters of ABa. The tagged gene is only expressed in the latter stages of the embryonic development. This tree is interactive in AceTree so the user can bring up the image corresponding to any point by clicking on the branch in question.

In such cases, AceTree offers several ways to view such information. In the normal viewing of images the red data is added to the red plane of the RGB ImageWindow so non-expressing nuclei show up in green as usual and expressing nuclei show up in yellow. The user can choose to view only green or only red as well. To make the red expression available in a more interpretable way, the lineage tree can be colored according to the extent of red expression. Figure 7 shows such a presentation for a data series where the tagged gene, *pha-4*, is known to be expressed in the pharynx and gut of the developing embryo including the E lineage and sublineages of MS and AB[11] (Murray, JI and Waterston, RH, unpublished).

**Figure 8:**
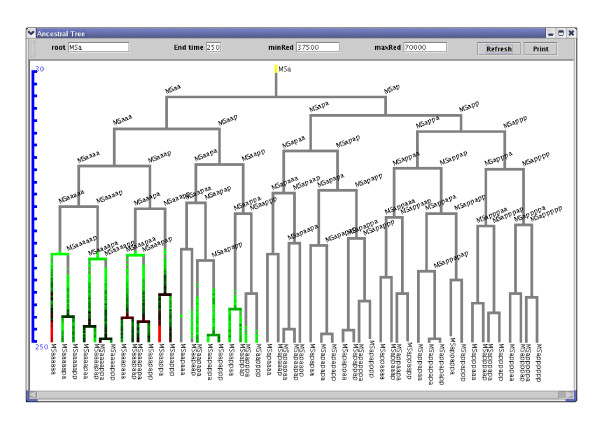
**Detailed view of a sublineage of interest**. To visualize the expression of the tagged gene in the MSa sublineage a vertical tree representation can be used. Progression of expression can be followed by the color scheme, which goes from green to black to red as the intensity of expression increases. Expression is detected in several daughters of MSaa but not in daughters of MSap.

Here, the tree branches are color coded to show the extent of red expression: if the cell is not expressing above the minimum threshold, the branch is shown in gray. For cells that are above the threshold the color starts at light green, getting darker, then black, then shades of red as the expression increases. In Figure 7, the entire lineage is captured and the overall character of gene expression is seen at a glance. The cell annotations are shown for every tenth leaf in the lineage. The user can adjust the spacing between lines and line thickness to explore things in more detail, but for detailed review, the tree version shown in Figure 8 may be more effective. Here, the MSa sublineage is shown and all cells are named. For further exploration, these trees are "live" like the JTree so a click on a branch brings up the image corresponding to that cell and time and makes it the "current cell" in the main control where the red expression value is reported numerically.

**Figure 9:**
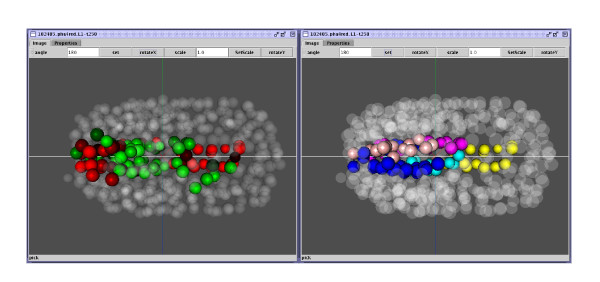
**Showing gene expression using the space filling model**. Two different views show the same time point, comparing the observed expression with that reported in the literature. These are ventral views with anterior on the left. The coloring scheme on the left characterizes the expression level of the tagged gene: cells expressing the gene are shown clearly with different shades of green and red using the scheme of Figures 7 and 8; non-expressing cells are grayed out. On the right, the sublineages reported in the literature to express the gene are shown with sublineage specific colors and cells not reported to be expressing are grayed out. The sublineage color scheme is: ABal in pink; ABar in blue; E in yellow; MSaa in magenta; MSap in cyan.

Figure 9 demonstrates how the space-filling model can be used to study gene expression in this situation. Two alternative views of the embryo at time 250, when there are about 500 cells present, are shown. The view on the left emphasizes the observed expression pattern; that on the right the expression pattern expected from the literature on the gene of interest. Both views are rotated so we see the ventral side with the anterior to the left and both use more advanced coloring features to bring out information.

On the left, each nucleus is colored according to the degree of red expression using the same scheme as in Figure 7 except that nuclei that are not expressing are shown in transparent white. Thus according to the data, all cells shown clearly are expressing the tagged gene, with the expression being especially high in those shown in red.

In the view on the right transparent white is again used, in this case for cells where, according to the literature, no expression is expected. Within the groups of cells that are reported to express the gene of interest, the lineage color scheme is: ABal in pink; ABar in blue; E in yellow; MSaa in magenta; MSap in cyan. Pairing the two 3D representations permits the investigator to consider the validity of the hypotheses about the location of gene expression in a very detailed way.

### Embryonic rotation

**Figure 10:**
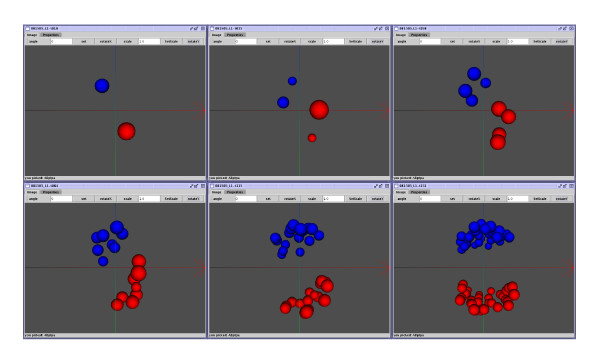
**Using the space filling model to track embryonic rotation**. The ABpl sublineage is shown in red; ABpr in blue; other cells are not shown. Each time point is just prior to the next round of cell divisions. A rotation of about 45 degrees of the axis between the two groups can be seen as development proceeds. Bear in mind that in a 3D view, cells closer to the viewer will appear larger than more distant cells.

Aspects of embryo morphology that are difficult to show in other ways can be vividly illustrated using tools available in AceTree. The pattern of rotation of the developing embryo illustrates this. During normal development, the embryo has been reported to undergo two rigid-body rotations around its a-p axis[1]. Variability in the direction and magnitude of this rotation was reported by Schnabel et al [3]. In Figure 10 a series of 3D images drawn from the annotations is shown as viewed from the anterior/posterior axis with the dorsal on top. Only cells from the ABp lineage are shown and the time points – 18, 35, 58, 84, 115, 151 – are just prior to the next round of divisions in the series. Cells from the ABpl sublineage are shown in red; ABpr in blue. A clockwise rotation of about 45 degrees can be visualized as development proceeds.

**Figure 11:**
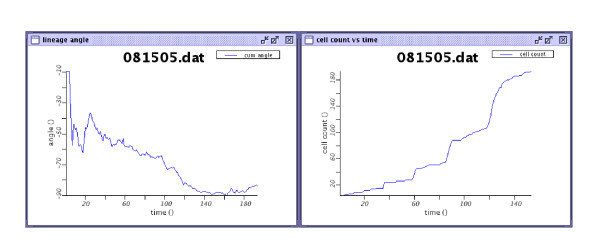
**Quantification of embryonic rotation**. The angle of the line connecting the centroids of the ABal and ABar sublineages is tracked verses time on the left. The cell count verses time is on the right. After a rough beginning, the angle increases relatively smoothly after time point 30.

One method of quantifying the angle is to visualize a line drawn between the center of mass of the ABpr cells and that of the ABpl cells. The angle of this line in the plane can then be plotted as shown in the left graph of Figure 11. The right side of the figure shows the total cell count in the embryo over time. The calculated angle increases clockwise relatively smoothly after the 15 cell stage of the embryo which occurred at about time 30 in the series.

## Conclusion

Bao et al [2] described the lineaging protocol and presented the results of a series of 20 lineaged embryos, using the low error rate observed to establish the validity of the overall approach. The minimum requirement for AceTree was that it facilitate curation and editing of the automated lineages. Bao *et al *reported that an earlier version of AceTree allowed curation and editing of a series through the 194 cell stage in about 2 hours and that an additional 2–8 hours were required to edit through the 350 cell stage. Editing time is a function of both the quality of the StarryNite output and the utility of AceTree. A goal for future versions of AceTree and StarryNite should be to further expedite editing beyond the 194 cell stage.

The longer-term goal of enhancing the researcher's capability of extracting biological significance from the data is more difficult to quantify. In this regard, AceTree is in a position similar to a number of software tools reported in the literature, especially those supporting the analysis of microarray data [12,13]. There, as here, the tools permit a combination of data clustering and tree representations, which are meant to guide the researcher through a complex dataset. In the final analysis, the clues obtained by using such tools form only a part of the evidence that leads to new knowledge. We are encouraged by the character of the examples shown here: the work with the *lit-1 *RNAi treated embryo; the visualizations of the tagged gene; the embryonic rotation study. We remain open to suggestions for additional features deemed helpful by practicing researchers.

## Methods

### User-interface classes

The AceTree class provides the main control and is the heart of the application. The link to StarryNite is established through the reading of the nuclei files, which are the main output of that program and by associating them with the same tiff images that were used in the automated lineaging. While this is essentially a user interface class, it is in control of cell and image navigation and as such contains code that embodies the essence of our application. The user navigates by a variety of methods: mouse clicks on the JTree, use of the cell selection panel, use of the button pad and its keyboard equivalents, mouse clicks on the AncestralTree trees, and the movie control. The "current cell" feature is controlled in AceTree. AceTree calls into play about 40 classes that handle various aspects of the user interface. These in turn make use of the javax.swing base classes.

The ImageWindow class makes the raw data of the process accessible and guides the eye by added annotation: circles locating the cells and the current cell, cell names, sister display. ImageWindow relies on classes of the ImageJ package for rendering the tiff images and annotating them. The EditImage class is derived from ImageWindow.

The Image3D class is built upon Java3D and this package is required in addition to Java 1.4.2 for the 3D feature to be available.

### Application data structures

The technical underpinnings of the application are embodied in five classes: Nucleus; NucleiMgr; Identity; Cell; AncesTree.

The Nucleus class contains the elements used to describe each nucleus at each time point. The member variables are all public and are directly accessed wherever needed in the AceTree code. The specific variables are those shown in Figure 2: identity, index, status, predecessor, successor1, successor2, x, y, z, size, green weight, red weight.

The NucleiMgr class holds the nuclei_record: a Vector of Vectors where the final elements are instances of the Nucleus class. Thus one Vector of the nuclei_record represents all nuclei at a time point. NucleiMgr has code for reading the nuclei files and creating its internal representation as well as code for recreating the successor indices after an edit operation (the "rebuild" action). The various edit operations act directly on the nuclei_record. AceTree supports saving an edited nuclei_record to nuclei files in the same form as the original files.

The Identity class implements the code to assign cell names. Identity is a singleton class and contains the key public function identityAssignment(), which generates names. Two naming methods described by Bao[2] are implemented. The main loop of STANDARD naming calls the private sisterID() function, which examines the parent/daughter orientation and attaches the implied character to the growing name. Canonically, the plane of division was determined by the orientation of the spindle axis at the initiation of division, with a preference given to the a/p division. Most divisions were assigned the addition of 'a' to one sister and 'p' to the other. In the current images the spindle is invisible. Instead, we rely on the axis of the daughter cells. When the x position of one sister can clearly be seen to be less than that of the other (say more than one quarter of a cell diameter), the daughter is assigned 'a'. Failing that, the code looks for a y axis difference and if that too is insufficiently large the z direction is used. Naturally there is some special case code in Identity to handle the germ line, polar bodies, and the EMS division.

Naming can proceed only when the orientation of the embryo is known. The imaging protocol guarantees that the x image axis will align with the anterior-posterior axis but cannot guarantee that the positive x direction is posterior. Likewise the dorsal-ventral and left-right axes may variously align with the y and z axes. The Identity module determines the axes by considering the 4 and 8 cell stages. ABa and ABp are distinguished from EMS and P2 by their earlier divisions and the initial axes can be unambiguously determined. If axes are not determined, all names are stylized: sublineages are called "Nuc" with a number added to distinguish the sublineage and directional letters to track subsequent divisions.

CANONICAL naming relies on a rule table based on the Sulston[1] lineage. The rule tells for each canonical parent what axis to use to examine the division and how the daughters are to be named to stay within the canonical list. Finally, there is "manual" naming for use in manual lineaging. The choice of naming method is a user option: canonical is the default.

The Cell and AncesTree classes enable AceTree to offer tree representations based on the annotation information in the nuclei files. The Cell class derives from the Java DefaultMutableTreeNode class. That class offers all the normal data structure features of trees such as the various traversals. AceTree calls the constructor of AncesTree giving it a NucleiMgr object. The constructor calls the private processEntries() function which proceeds through the nuclei_record from time point to time point. In the general case, the presence of a new cell is detected by examination of the successor indices and new Cell objects are created for each sister. When AceTree needs to refer to the tree as a whole, it refers to the root Cell: the properties of the DefaultMutableTreeNode take care of the details.

Access to cells within AceTree is facilitated by two hashtables kept by AncesTree and made available via access functions: iCells and iCellsByName. The member variables of the Cell object include: name, starting time, ending time, ending fate (alive, divided, died) and a hashKey which is constructed from the time and index of its Nucleus at birth. The Cell object also contains the drawing code needed to render it in Ancestral Tree style trees.

## Availability and requirements

AceTree is available as part of the StarryNite-AceTree package from . The AceTree.jar file contains the source code. It requires Java 1.4.1 and the ImageJ jar file ij.jar; Java3D must be installed to use the space filling model features. AceTree has been tested on linux, macintosh, and windows. The AceTreeDemo download contains the program and a data set in a single zip file of about 200 MB. The AceTree Help menu provides a tutorial in html called AceTreeDemo, which can be followed to learn the program features, the character of the data, and the power of the annotations developed by StarryNite. AceTreeDemo.html can be extracted from the jar file and printed from a browser to provide an offline manual. The AceTree.jar file in the demo package is the full featured program and can be used with other data available on the site.

## Authors' contributions

TJB designed the software with input from all authors. TJB programmed the software. Experiments were conceived and performed by ZB, JIM, and CA. TJB wrote the manuscript with significant input from ZB, JIM, and RHW.
